# Onychomycosis due to* Candida parapsilosis* in a Child with Ventricular Septal Defect: An Unusual Predisposition

**DOI:** 10.1155/2016/7026068

**Published:** 2016-04-18

**Authors:** Supram Hosuru Subramanya, Deependra Hamal, Niranjan Nayak, Shishir Gokhale

**Affiliations:** Department of Microbiology, Manipal College of Medical Sciences, Pokhara 33700, Nepal

## Abstract

*Candida parapsilosis* is emerging as a potential pathogen for onychomycosis. A 4-year-old male child with perimembranous ventricular septal defect (VSD) was admitted with features of cystitis and was treated with broad spectrum antibiotics. Two weeks later, he developed yellowish discoloration of nails of both hands. The sloughed out nail, on microscopy, showed numerous yeast forms that were identified as* Candida parapsilosis* by both phenotypic and genotypic methods. Antifungal sensitivity testing of the isolate was performed by microbroth dilution method in accordance with CLSI guidelines. Patient was successfully treated with topical amphotericin B and oral fluconazole. Thus, one should have a high index of suspicion of* C. parapsilosis* onychomycosis, especially when the patient is in the paediatric age group, presenting with unusual predisposing condition like congenital heart disease, and is on broad spectrum antibiotics.

## 1. Introduction

Onychomycosis is an infection of the nail caused by dermatophytes, yeasts, and other molds. According to some investigators, onychomycoses can comprise 30% of all superficial fungal infections and up to half of all nail disorders [1]. Onychomycosis predominantly affects adults, especially individuals more than 50 years of age, because an increase in nail plate thickness and a decrease in nail growth rate make these subjects more vulnerable to such infections [2]. Most common etiological agents of onychomycosis are dermatophytes such as* Trichophyton rubrum*, followed by* T. interdigitale* and* T. mentagrophytes* [2, 3]. The role of* Candida* species in onychomycosis has also been established [4].* Candida* is still largely considered to cause onychomycosis secondary to paronychial disease or peripheral vascular disease [4–6]. According to earlier studies, out of all* Candida* species,* C. albicans* accounted for 57–87% of the infections [5, 7].* Candida parapsilosis* was known to be occasionally responsible for pathological lesions of the nails [5] but is now emerging as one of the important etiological agents of onychomycosis. Though* C. parapsilosis* nail infection is commonly reported in association with other fungal pathogens, we report here a case of onychomycosis due to* C. parapsilosis* alone with some unusual predisposing conditions.

## 2. Case Report

**Figure 1:**
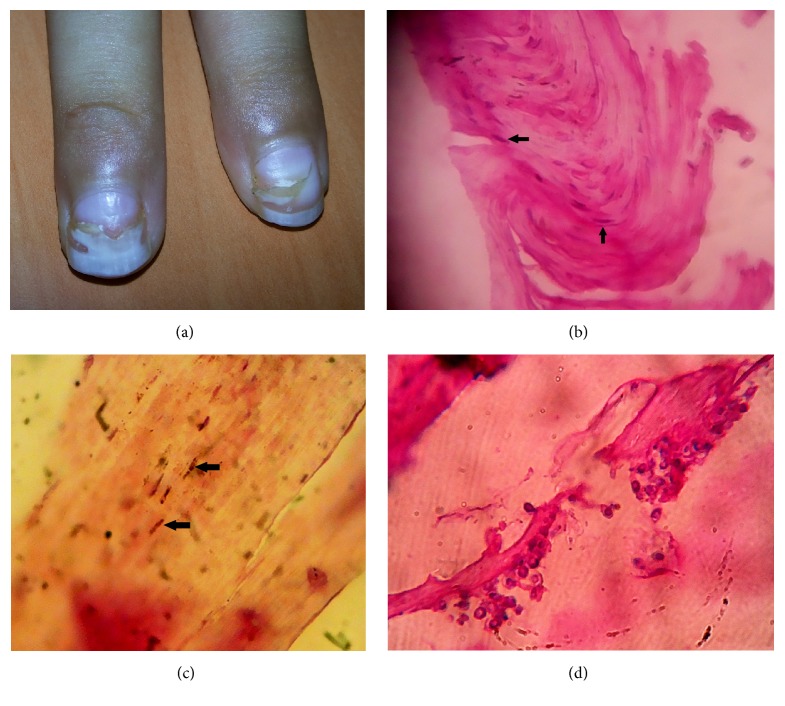
(a) Thickened, discolored, and sloughed out nail; (b, c) the H&E preparations showing thin hyphae (arrow head); (d) another H&E preparation that shows yeast cells with coarse pseudomycelia (giant forms).

A 4-year-old young male child was admitted to Manipal Teaching Hospital (MTH), Pokhara, Nepal, with the complaints of fever and chills of four days' duration and difficulty in micturition since 2 days. The parents noticed occasional discharge from the urethral meatus and passage of fresh blood at the end of micturition since 2 days. On examination, the patient's general condition was fair. His temperature was 98.6°F, respiration rate was 28 per minute, and heart rate was 102 per minute. There was no pallor, no icterus, and no lymphadenopathy. There was mucoid discharge from the urethra with mild congestion at the preputial area. The case was diagnosed as urethrocystitis. Both the urethral discharge and the urine specimens were sent for culture and sensitivity testing on the second day of admission. Urine sediment showed plenty of pus cells. On culture, it grew* Escherichia coli* [>10^5^ CFU/mL] which was sensitive to norfloxacin, ciprofloxacin, gentamicin, ceftazidime, cefotaxime, nitrofurantoin, piperacillin, cefoperazone-sulbactam, and netilmicin and resistant to cephazoline and ampicillin. The urethral discharge also grew* E. coli* with sensitivity pattern similar to the urinary isolate as noted above. On day four of admission, patient was started on cefixime injection 750 mg 8 hourly for 5 days and gentamicin injection 80 mg OD for 5 days, along with paracetamol syrup 7.5 mL SOS. Patient became afebrile three days after the administration of antibiotics. During this time, cardiovascular system examination revealed a pansystolic murmur over the left parasternal region, and hence he was referred to cardiology for consultation. He was diagnosed as a case of perimembranous ventricular septal defect (VSD) with small left to right shunt. There was no atrial septal defect nor was there any patent ductus arteriosus. Since there was recurrence of fever, the child was kept on the same antibiotic regime. However, fever subsided after another seven days of treatment and the patient was discharged with the advice for cardiology review after 6 months. One week later, parents noticed that the child had developed yellowish discoloration of the nails of both hands (Figure 1(a)). He was again brought to MTH for dermatology consultation. On examination, the nails of both hands were painless with sloughing of the distal parts of the nails. Nail clippings from the sloughed out area were collected with aseptic precautions. Direct microscopy under 20% KOH wet mount of the clippings showed numerous yeast cells. A small fragment was processed for histopathological examination using haematoxylin and eosin stain which demonstrated fungal elements, yeast cells with coarse pseudomycelia (Figures 1(b), 1(c), and 1(d)). Sample was cultured on 2 sets of Sabouraud's Dextrose Agar slants with chloramphenicol which were incubated at 25°C and 37°C. The media at 37°C showed dull white creamy moist colonies after 3 days' incubation. The organism was identified as* Candida parapsilosis* by conventional methods [8] and confirmed by sequencing the D1-D2 portion of the 28S rRNA gene [9]. Antifungal sensitivity testing of the isolate performed by micro-broth dilution method in accordance with CLSI guidelines [10] showed the following MICs (*μ*g/mL) for various antifungal agents tested: fluconazole (4), voriconazole (0.1), anidulafungin (0.3), caspofungin (0.1), micafungin (4), amphotericin B (2), itraconazole (0.3), and posaconazole (0.1). The patient was successfully treated with topical amphotericin B and oral fluconazole 6 mg/kg to start with, followed by 3 mg/kg from day two onwards for 6 weeks with marked improvement in skin discoloration and restoration of vascular tissue in the nail bed.

## 3. Discussion

Onychomycosis is not an infrequent clinical problem at the extremes of ages, irrespective of the immune status of the patients [5–7]. In the present case, at least two risk factors for* C. parapsilosis* nail infection were observed: the patient had received broad spectrum antibiotics for more than 2 weeks and secondly the child had VSD. It is well established that children with unrecognised VSD may have clubbing [11] which may go unnoticed and may give rise to destruction of the skin barrier caused by minor trauma with subsequent alteration in the quality of the nail and dystrophy of the nail [12]. The role of nail dystrophies in relation to diseases such as Systemic Lupus Erythematosus (SLE) and diabetes mellitus and prolonged use of corticosteroids have previously been reported [13]. Hay et al. noticed three patterns of nail diseases due to* Candida*: total dystrophic onychomycosis, mostly seen in chronic mucocutaneous candidiasis; proximal and lateral nail dystrophy, secondary to chronic paronychia; and distal and lateral nail dystrophy, associated with onycholysis, sloughing of the nail with peripheral vascular disease, and finger and toe nail abnormalities [4]. The present case with distinctive features of sloughed out nails and melanonychia in the distal parts of the nails could belong to the third category described above.

*Candida parapsilosis*, being a common inhabitant of the skin, was often regarded, in the past, as a contaminant or mere colonizer in many clinical specimens [8]. As previously documented,* C. parapsilosis* colonizes the surface of nails after prior invasion by other* Candida* species [13]. In the present case, however,* C. parapsilosis*, alone caused the nail pathology. This is in agreement with the observations of Gautret et al. [14] who reported nail dystrophy and melanonychia due* to C. parapsilosis* in an elderly patient who had previous history of crushing injury in his hand. Earlier reports have suggested that* C. parapsilosis* could be the most prevalent* Candida* species in both finger nail and toe nail infections [1, 13]. The clinical presentation in our case was not typical of those described in the literature [4, 8]. Since the child had involvement of several nails, topical amphotericin B and oral fluconazole was initiated and the condition improved satisfactorily.

## 4. Conclusion

High index of suspicion of* C. parapsilosis* onychomycosis, especially when the patient is in the paediatric age group presenting with unusual predisposing condition like congenital heart disease and broad spectrum antibiotic therapy must be entertained. Clubbing and other nail abnormalities presumably lead to nail dystrophy and subsequent colonization by* C. parapsilosis*.
